# Global transcriptome profile of the developmental principles of in vitro iPSC-to-motor neuron differentiation

**DOI:** 10.1186/s12860-021-00343-z

**Published:** 2021-02-18

**Authors:** Emilia Solomon, Katie Davis-Anderson, Blake Hovde, Sofiya Micheva-Viteva, Jennifer Foster Harris, Scott Twary, Rashi Iyer

**Affiliations:** 1 Los Alamos National Laboratory, Bioscience Division, Los Alamos, NM USA; 2 Los Alamos National Laboratory, Analytics, Intelligence, and Technology Division, Los Alamos, NM USA

**Keywords:** iPSCs, Motor neurons, Stem cell reprogramming, Transcriptomics, Neuronal development, In vitro neuronal networks

## Abstract

**Background:**

Human induced pluripotent stem cells (iPSC) have opened new avenues for regenerative medicine. Consequently, iPSC-derived motor neurons have emerged as potentially viable therapies for spinal cord injuries and neurodegenerative disorders including Amyotrophic Lateral Sclerosis. However, direct clinical application of iPSC bears in itself the risk of tumorigenesis and other unforeseeable genetic or epigenetic abnormalities.

**Results:**

Employing RNA-seq technology, we identified and characterized gene regulatory networks triggered by in vitro chemical reprogramming of iPSC into cells with the molecular features of motor neurons (MNs) whose function in vivo is to innervate effector organs. We present meta-transcriptome signatures of 5 cell types: iPSCs, neural stem cells, motor neuron progenitors, early motor neurons, and mature motor neurons. In strict response to the chemical stimuli, along the MN differentiation axis we observed temporal downregulation of tumor growth factor-β signaling pathway and consistent activation of sonic hedgehog, Wnt/β-catenin, and Notch signaling. Together with gene networks defining neuronal differentiation (neurogenin 2, microtubule-associated protein 2, Pax6, and neuropilin-1), we observed steady accumulation of motor neuron-specific regulatory genes, including Islet-1 and homeobox protein HB9. Interestingly, transcriptome profiling of the differentiation process showed that Ca^2+^ signaling through cAMP and LPC was downregulated during the conversion of the iPSC to neural stem cells and key regulatory gene activity of the pathway remained inhibited until later stages of motor neuron formation. Pathways shaping the neuronal development and function were well-represented in the early motor neuron cells including, neuroactive ligand-receptor interactions, axon guidance, and the cholinergic synapse formation. A notable hallmark of our in vitro motor neuron maturation in monoculture was the activation of genes encoding G-coupled muscarinic acetylcholine receptors and downregulation of the ionotropic nicotinic acetylcholine receptors expression. We observed the formation of functional neuronal networks as spontaneous oscillations in the extracellular action potentials recorded on multi-electrode array chip after 20 days of differentiation.

**Conclusions:**

Detailed transcriptome profile of each developmental step from iPSC to motor neuron driven by chemical induction provides the guidelines to novel therapeutic approaches in the re-construction efforts of muscle innervation.

**Supplementary Information:**

The online version contains supplementary material available at 10.1186/s12860-021-00343-z.

## Background

Human neuronal tissue lacks regenerative capacity, leaving few treatments available following neuronal injury or neurodegeneration. In the past decade, an interest in direct neuronal reprogramming of stem cells into motor neurons (MNs) has emerged as a solution to generate human neuronal tissue for therapeutic applications. MNs form synapses to potentiate electrical signals from the CNS into peripheral tissues. They play a critical role in the formation of neuromuscular junctions (NMJs), where MN axons terminate on muscle fibers and neurotransmitters are released to trigger muscle contractions. NMJs are cholinergic synapses, where the neurotransmitter acetylcholine (ACh) is released from the presynaptic MN terminal for uptake by postsynaptic ACh receptors on the target muscle cell [1]. This critical function is disrupted in neurodegenerative motor neurons diseases like as Amyotrophic Lateral Sclerosis (ALS).

Several in vitro protocols have been developed to convert progenitor cells, such as human inducible pluripotent stem cells (iPSCs), into MNs [2–4]. There are still challenges limiting clinical application of these iPSC-derived MNs. For example, the generation of physiologically active neurons requires a lengthy cell maturation period and often results in a heterogeneous population of neuronal subtypes [5]. Protocol reproducibility can also vary as different cell lineages have unique maturation and functional properties. To address these challenges, we present a 28-day transcriptome study coupled with functional assays. Our main objective was to resolve the underlying mechanisms driving MN differentiation. The results from this study can guide experimental strategies to obtain populations highly enriched with the desired MN subtype.

Here, we followed an established protocol [6] to differentiate iPSCs into MNs using chemically defined media conditions. Efficient neural conversion is based on mimicking in vivo neurogenesis where extrinsic and intrinsic signals are introduced in culture, yielding a relatively pure MN population [7, 8]. During neuronal differentiation, there are cascading effects as signaling pathways activate transcription factors to upregulate expression of MN specific genes. Neural induction of iPSCs is driven by simultaneous inhibition of tumor growth factor-β (TGFβ), activin, Nodal, and bone morphogenic protein (BMP) signaling. Similar to processes that occur during early development, inhibition of those signaling pathways promotes differentiation along the neuronal lineage primarily through inhibition of pluripotency and blocking alternative lineage differentiation. Several other pathways, including the Wnt signaling pathway, regulate neuronal differentiation. The protocol implemented in this study included three core chemical compounds to inhibit TGFβ and BMP signaling pathways and simultaneously activate Wnt signaling. Following neural induction of iPSCs, neuronal progenitor cells were patterned with all-trans retinoic acid (RA), to promote caudal (spinal cord) identity, and ventralization was promoted by activation of Shh signaling with Purmorphamine. Finally, synchronization of the maturation process, through elimination of dividing cells, was aided by inhibition of the Notch signaling pathway resulting in mature MNs.

Genome-wide transcriptome studies provide in-depth knowledge of regulatory pathways that shape cellular morphology and function. Such information is crucial for the design of novel neuron regenerative therapies and cell-based drug discovery platforms. Previous studies based on single cell transcriptomics of Amyotrophic Lateral Sclerosis (ALS) patient-derived iPSCs discovered the underlying mechanisms of disease pathology [9] and the regulatory dynamics of MN differentiation [10]. Others have investigated the iPSC-derived MN axonal transcriptome and found key regulatory pathways presenting potential drug targets for treatment of genetic disorders [11]. A detailed transcriptomics study by Burke et al. proposed an influence of the genetic background of the iPSC donors on each pivotal step of iPSC-initiated corticogenesis [12]. Further, single cell RNA-seq analysis of iPSC-derived spinal MN demonstrated that in vitro differentiation does not produce a homogeneous MN population [8].

Here we performed a comprehensive transcriptomic analysis of in vitro iPSCs-derived MNs to characterize the key principles of MN development by analyzing bulk transcriptome data from five crucial time points of MN neurogenesis: iPSCs (D0), NSCs (D7), post-mitotic MNP cells (D13), eMNs (D18), and mature MNs (D28). We interrogated the transcriptomic signatures using next-generation RNA-sequencing (RNA-Seq) technology and performed in situ validation of key pluripotency and MN specific biomarker expression. Additionally, we characterized the functionality of iPSC-derived MNs via electrophysiological analysis of neuronal network connectivity. Our results corroborate transcriptomic profiles previously identified in the astrocyte-to-neuron transformation process [3]; specifically, upregulation of Shh and Wnt signaling pathways. We observed altered gene expression of TGFβ signaling components during the early stages of iPSC conversion into neural stem cell (NSC) in response to chemical inhibition. However, we detected an upregulation of positive TFGβ regulators in the subsequent steps of neurogenesis.

By applying a LANL-developed Ontology Pathway Analysis software (OPaver) we found that calcium (Ca^2+^) signaling through cyclic adenosine monophosphate (cAMP) and LPC is downregulated during the conversion of the iPSC to NSC and remains silenced until the final stage of MN maturation when the regulatory pathway becomes a driving force for the neuronal synaptic activity. Another key finding is that during the in vitro development of MN in 2D monocultures, ionotropic nAChR are expressed in the early MN stage and are subsequently replaced by the G protein coupled receptors (GPCR) -type muscarinic AChR in mature MN, probably due to lack of metabolic stimuli that are naturally released by the muscle tissue in vivo. While our findings provide unique insights into the temporal mechanism of iPSC-derived MNs they also indicate the advantages of using a co-culture system, of MN and muscle, to enable the development of physiologically relevant MNs types akin to that obtained in vivo.

## Results

### A four-step process of iPSC differentiation into MNs

**Fig. 1:**
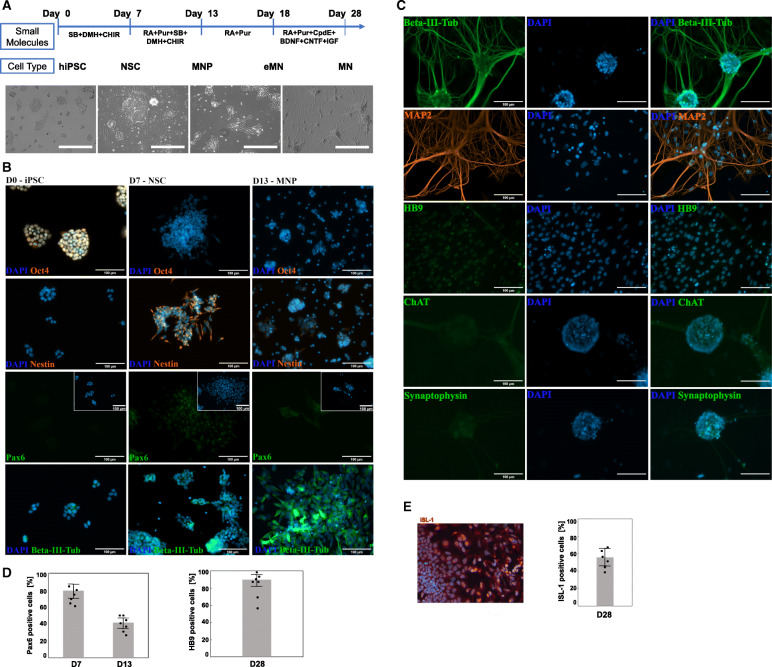
Differentiation of WTC-11 induced pluripotent stem cells (iPSC) into motor neurons (MN). **a** Schematic diagram of the overall experimental design. Shown are the small molecule stimuli for each developmental stage and the corresponding morphological changes in the course of iPSC conversion into MN. The scale bar for the phase contrast images is 400 μm; (**b**) Representative immunofluorescence images of key pluripotency (Oct4) and pan-neuronal (Nestin, Pax6, and β-III tubulin) biomarkers indicated at efficient iPSC differentiation into neuronal stem cells (NSC) at day 7th post-induction (accumulation of Nestin is shown in orange). Upregulation of Pax6 and β-III tubulin (shown in green) at day 13 of iPSC induction marked the formation of motor neuron progenitor (MNP) cells. **c** Immunostaining of neuronal structural proteins (beta-III-tubulin in green, MAP2 in orange), motor neuron specific markers (HB9 and ChAT in green), and synaptic vesicles protein (Synaptophysin in green) showed a pure population of mature MN at day 28 post-stimulation of iPSC. **d** Image quantification of Pax6- and HB9-labeled cells exemplified the percentage of cells that transitioned from NSC to MN. **e** Immunocytochemistry with anti-ISL1 antibody and quantitative analysis of biomarker positive cells at D28 of the differentiation protocol. The graphs show average of 6 cultures with > 300 cells in random fields for each culture. Scale bar: 100 μm

The initiating step for iPSC differentiation to neuronal stem cells (NSC) is driven by inhibition of TGFβ, activin, nodal, and BMP signaling pathways while simultaneously activating the Wnt signaling pathway to sustain cell proliferation. Therefore, to convert undifferentiated iPSCs into a NSC lineage the culture media was supplemented with SB431542 (SB) and DMH-1, inhibitors of activin receptor-like kinases (ALK4, ALK5, ALK7, and BMP), and CHIR99021 (CHIR), a Wnt pathway activator. MNPs and early motor neurons (eMNs) were patterned through activation of the Shh pathway by Purmorphamine (Pur). The final step of MN maturation and specification was aided by a Notch signaling pathway inhibitor, Compound E (CpdE), to synchronize the maturation process through the elimination of dividing cells. Neurotrophic and growth factors were supplemented to facilitate neuronal growth and maturation (Fig. 1a).

Within a 13-day period, we observed changes in cell morphology, including a gradual reduction in the cell soma and multiple extensions of thin neurites upon iPSCs conversion into MNPs. Furthermore, we monitored protein accumulation of tissue-specific markers via immunohistochemistry staining (Fig. 1b). Consistent with the morphological features exemplified by high nuclear-to-cytoplasmic ratio and compact multilayer colonies [13], the undifferentiated iPSCs expressed a key pluripotency marker, Oct4 (Fig. 1b; D0). We noted the formation of NSCs at D7 by the disappearance of Oct4 and the accumulation of Nestin and Pax6. Nestin, implicated in cell division and radial axon growth [14, 15], was downregulated by D13 which coincided with increased expression of the pan-neuronal filament protein class III β-Tubulin (βIII-Tub). Cells entered the MNP developmental stage on D13 and transformed to eMNs on D18 when both Nestin and Pax6 expression was significantly downregulated (Fig. 1b, d). Finally, we detected mature MNs featuring high levels of the structural proteins, βIII-Tub and microtubule-associated protein 2 (MAP2); and increased accumulation of MN specific markers: MN homeobox protein (HB9), choline acetyltransferase (ChAT), and the synaptic vesicles protein (Synaptophysin) (Fig. 1c, d). HB9 is an essential transcription factor and early marker of cholinergic neurons [16] and ChAT is an enzyme required for the synthesis of the neurotransmitter acetylcholine (ACh). The changes in morphology were pronounced as dendrites extended and expressed MAP2, a neuron-specific cytoskeletal protein critical for successful projection of dendrites [17, 18]. Accumulation of the pan-MN marker ISL-1 (~ 60% ± 12%, Fig. 1e) together with the HB9 transcription regulator (~ 85% ± 15%, Fig. 1d) at D28 of the iPSC differentiation indicated at formation of cell populations enriched in mature MNs.

### MNs form functional network connections

**Fig. 2:**
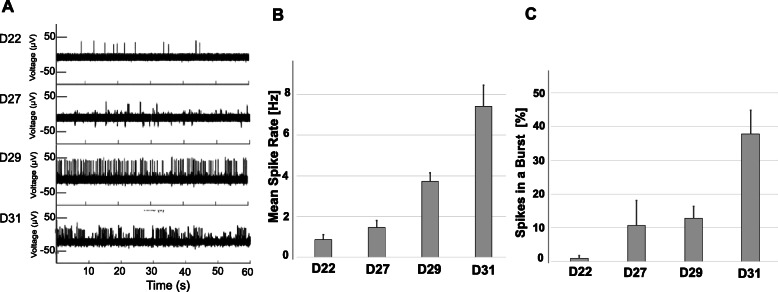
Electrophysiological analysis of (MNs). **a** Time-dependent increase of spontaneous action potential (AP) spikes generated by MNs and recorded from a representative electrode (one of 144) on a multi-electrode (MEA) chip. MN were plated on the MEA chip at 18 days post-chemical induction of iPSC (D18) and electrophysiological activity was recorded at the indicated time points of MN maturation (D22-D31) for 1 min. The frequency of AP spikes (**b**) and the percentage of spikes in the burst **(c**) dramatically increased within the neuronal networks over time. Shown are the mean and standard error from 12 electrodes at each sampling day post iPSC induction

MN activity was characterized by multi-electrode array (MEA) recordings of cellular electrophysiological responses. This non-invasive method allows for repetitive recordings of spontaneous electrical firings at various times during neuronal differentiation. Spontaneous oscillations in extracellular action potential (AP) were recorded after day 20 of neuronal differentiation, and the AP frequency increased the longer the cells differentiated on the MEA (Fig. 2a). By day 31 (D31) the firing patterns became more organized in highly synchronous bursts of network activity. The AP spike rate increased as differentiation progressed (Fig. 2b). The increase in the number of bursts, and percentage of spikes in bursts, signified successful formation of synaptic connections with synchronized AP firings (Fig. 2c).

### Unbiased transcriptome analysis of chemically stimulated iPSC differentiation into MNs

**Fig. 3:**
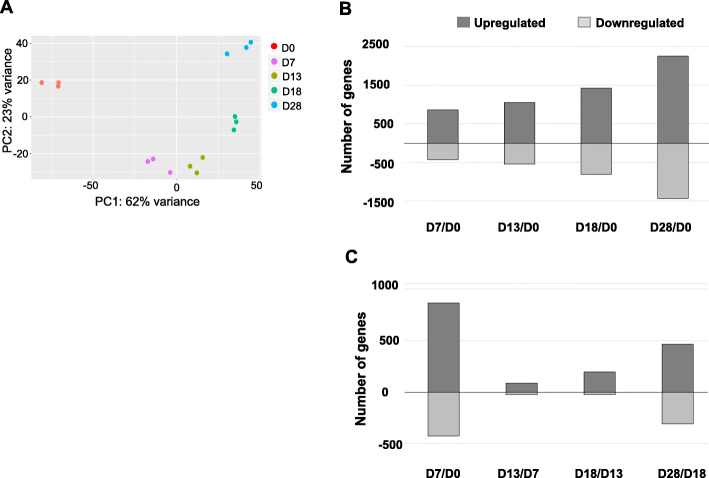
Transcriptome analysis of cellular responses to MN maturation. **a** Principal component analysis of all samples, *n* = 3; **(b**) Histogram of differentially expressed genes (DEGs) (adjusted *p* < 0.0002, fold change > 3) among D7 - D28 samples in the pairwise comparison with D0; (**c**) Histogram of DEGs in pairwise comparisons between adjacent timepoints (D0-D7, D7-D13, D13–18, D18–28)

We performed whole-genome transcriptome analysis on RNA-Seq platform to explore gene regulatory pathways. Three independent biological replicates were collected and analyzed at each stage: iPSCs, NSCs, MNPs, eMNs, and MNs. The principal component analysis (PCA) of global transcriptome data revealed tight clustering of all replicates, indicating high reproducibility of gene expression profiles for each stage of development. Differentiation timepoints showed distinct genetic programs, as evidenced by the PCA variances (Fig. 3a). Applying pair-wise analyses, we further compared the differential gene expression profiles between cell populations at various developmental stages. The number of differentially expressed genes (DEGs) increased at each timepoint throughout the differentiation process when compared to D0, illustrating the steady transformation of iPSCs into MNs driven by chemical stimuli (Fig. 3b and list of DEG in Additional file 1). The greatest number of DEGs occurred upon the initial culturing of iPSCs in neural differentiation media supplemented with an inhibitor of TGFβ signaling pathway and a Wnt pathway agonist (Fig. 3c; D7-D0, see Additional file 1).

**Fig. 4:**
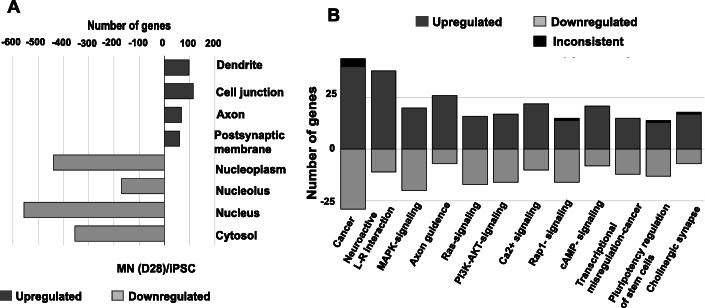
Bioinformatics analysis of transcriptomic data. **a** Gene ontology (GO) analysis of iPSCs and MNs as determined by the NCBI Database for Annotation, Visualization and Integrated Discovery (DAVID). The top four significant GO terms of upregulated and downregulated genes are listed, ranked by *p*-value, when comparing D0 iPSCs and D28 MNs. **b** OPaver analysis of 12 KEGG pathways ranked by the number of DEG during the chemical reprogramming of iPSCs into MNs: Pathways in cancer; Neuroactive ligand-receptor interaction; MAPK-signaling pathway; Axon guidance; Ras signaling pathway; PI3K-Akt signaling pathway; Calcium signaling pathway; Rap1 signaling pathway; cAMP signaling pathway; Transcriptional mis-regulation in cancer; Singling pathways regulating pluripotency of stem cells; and Cholinergic synapse

We analyzed RNA-Seq data with the NCBI Database for Annotation, Visualization and Integrated Discovery (DAVID) v6.8 [19] which categorized DEGs between D0 and D28 by gene ontology (GO) terms (Fig. 4a). Of the upregulated genes, 2118 were recognized by DAVID and 1683 (79.5%) mapped to specific GO terms consistent with a shift from undifferentiated mitotic cells (iPSC) to differentiated cells of the neuronal lineage. The upregulated DEGs were associated with GO terms specific to neuronal development: 98 genes were related to dendrites; 144 genes were involved in cell junctions, 68 genes were associated with axon formation, and 61 genes were involved in the postsynaptic membrane. Of the genes downregulated on D28 versus D0, DAVID recognized 1361 genes of which 1234 (90.7%) mapped to a specific GO. The downregulated genes were characteristic of cells undergoing active DNA replication, transcription, and translation associated with nucleoplasm (440 genes), nucleolus (170 genes), nucleus (556), and cytosol (354) GO terms (Fig. 4a).

LANL-developed OPaver [20] identified 12 gene signaling pathways that were significantly (*p* < 0.05) altered during iPSC conversion to MNs (Fig. 4b). The top 5 KEGG pathways represented genes involved in cancer regulation, axon guidance, calcium signaling, PI3K-Akt signaling, and MAPK-signaling pathways (Fig. 4b). The OPaver analysis further highlighted the critical role of membrane proteins in cell-to-cell interactions for neuronal development; specifically, neuroactive ligand-receptor interactions and the development of cholinergic synapses.

**Fig. 5:**
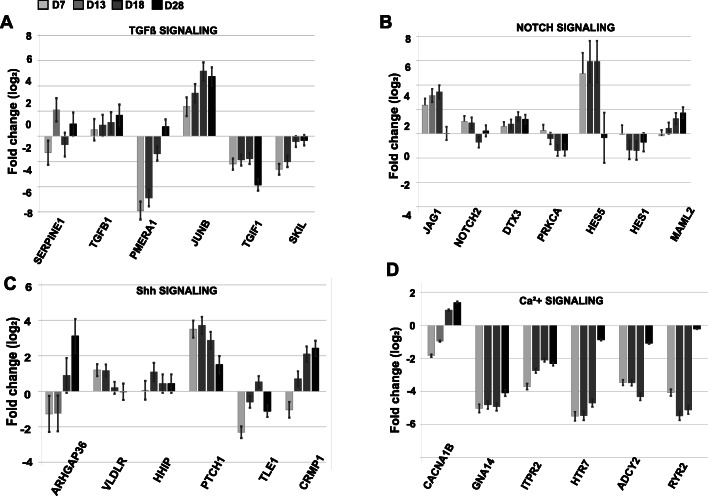
Differential gene expression in key signaling pathways shaping MN differentiation. Gene transcript levels were determined by global RNA-seq analysis of (**a**) TGFβ, (**b**) Notch, and (**c**) Sonic Hedgehog (Shh) pathways and (**d**) Calcium signaling. Fold change was calculated relative to RNA reads in iPSC (D0). The statistics (average value and standard error) were derived from three independent biological replicas with *p* < 0.001 determined by R-statistical analysis package

By focusing our analysis on cellular development pathways, we found significantly altered gene expression profiles of genes involved in TGFβ, Notch, and Shh signaling pathways (Fig. 5). Contrary to our expectations, 7 days after chemical inhibition of TGFβ, we observed a significant downregulation of genes acting as negative regulators of TGFβ signal transduction: *PMEPA1* and *TGIF1*, repressors of SMAD2 function, and *SKIL* the nuclear repressor of TGFβ-responsive genes [21]. At the same time, a gene positively regulated by TGFβ signaling, *JUNB*/*AP1*, was upregulated (Fig. 5a). These data indicate there was a transient inhibition (lasting less than 7 days) of the TGFβ signaling pathway, when small molecules were present in the culture media to target ALK receptor kinases. In the Notch signaling pathways, we observed a downregulation of *Hes1*, *NOTCH2*, and *PRKCA* after NSCs conversion into MNPs (Fig. 5b; D13). Interestingly, gene expression of *Hes5* and *JAG1* was upregulated until D18 when cells entered the eMN developmental stage. The final MN maturation phase required the addition of CpdE, a Notch signaling pathway inhibitor [22], along with growth factors: insulin-like growth factor 1 (IGF-1), brain-derived neurotrophic factor (BDNF), and ciliary neurotrophic factor (CNTF). Our results indicated that downregulation of Notch regulators *JAG1* and *Hes5* gene expression was required for the maturation of MNs (Fig. 5b; D28). Shh signaling is essential for patterning and specification of neuronal cells. Significant activation of a key Shh receptor gene, *PTCH1*, was observed at D7 prior to the addition of Pur, and remained upregulated throughout the differentiation process but decreased in MNs (D28), indicating that this gene may act as a positive regulator of cell division in human neuronal cells. In contrast, *ARHGAP36* and *CRMP1* genes were downregulated upon transition of iPSCs to NSCs (D7) and responded to Pur with gradual stimulation of gene expression in MNs at D18–28 (Fig. 5c).

### Validation of RNA-Seq data via RT-qPCR

**Fig. 6:**
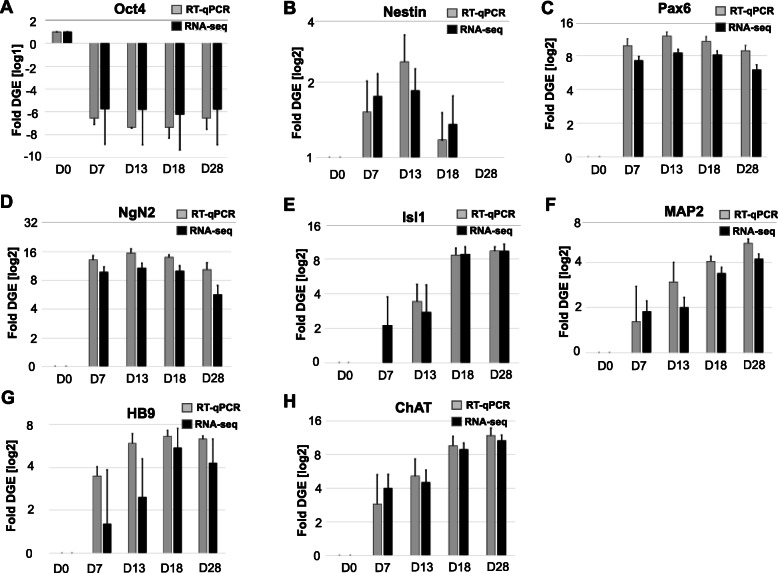
Comparative analysis of differential gene expression of key tissue development markers by RT-qPCR (gray) and RNA-seq (black). Pluripotency and NSC markers validated include (**a**) Oct4, transcription factor that maintains self-renewal and pluripotency; (**b**) Nestin, a filament protein marker of neural stem cells; (**c**) Pax6, a transcription factor that drives neurogenesis; and (**d**) NgN2, a neuronal-specific transcription factor. Motor neuron specification markers validated include (**e**) Isl1, a transcription factor required for motor neuron generation; (**f**) Map2, a neuron-specific cytoskeletal protein; (**g**) HB9, an early marker of cholinergic neurons; and (**h**) ChAT, an enzyme required for acetylcholine synthesis. Shown are the averages and standard error from three independent biological replicas from the iPSC to MN differentiation trajectory. The RNA-Seq transcripts were normalized to the total read per analyzed sample (in FPKM: fragments per kilobase per million mapped fragments) and the transcript levels determined by RT-qPCR were normalized to GAPDH as the endogenous sample control. Fold change was calculated for each developmental stage relative to transcript levels in iPSC (D0). Statistical significance (*p* < 1.5e-6) was determined with Student t-test

Applying RT-qPCR we further validated a subset of key gene markers of pluripotency, NSCs, MNPs, and MNs which were found to be differentially expressed by RNA-Seq profiling. Both methods detected the rapid decrease of pluripotency marker *Oct4* by D7 and increase of *Nestin*, *Pax6*, and *NgN2* expression reaching peak levels at D13 (Fig. 6a-d). Markers of MN specification (*Isl1*, *HB9*, *MAP2*, and *ChAT*) increased throughout the course of neuronal maturation (Fig. 6e-f, h). *ChAT* expression levels gradually increased throughout the differentiation process from NSCs to MNs (Fig. 6h). Collectively, quantitative analysis of gene expression via RNA-Seq closely correlated with RT-qPCR data showing dynamics of gene activities typical for the conversion of pluripotent cells to MNs. We observed transient activation of *Nestin*, a key regulator of cytoskeletal dynamics and cell division in NSC, and stable expression of pan-neuronal markers *Pax6*, *NgN2*, and *MAP2*. Our data indicated there was gradual accumulation of gene transcripts responsible for the synthesis of neurotransmitter, *ChAT*, and transcription factors, *Isl1* and *HB9*, whose concerted actions shape the phenotype and physiological activity of MNs.

### Cell signaling pathways driving iPSC conversion to MNs

The GO analysis, conducted by OPaver, identified 12 pathways that included a high number of DEGs as iPSCs were differentiated into MNs. We further investigated the DEG pattern of individual genes in the pathways listed below which had a critical role in shaping neuronal development and function (Tables 1, 2, 3, 4, 5). In each description below, ‘upregulated’ and ‘downregulated’ gene expression refers to a significance of *p* < 0.05 and log_2_ fold change ≥2 between sampling timepoints.

**Table 1:** DEGs identified in KEGG pathway: MAPK-signaling. Differential expression indicates the average log_2_-fold change in RNA-Seq transcript levels from three independent experiments at each sampling period: D7 vs D0, D13 vs D7, D18 v D13, and D28 v D18. The *p*-values were ≤ 0.001. Negative log_2_-fold change corresponds to gene downregulation and the positive values indicate gene activation

MAPK-signaling pathway			
Gene ID	Description	Differential expression	Time
CACNA1B	voltage-dependent calcium channel N type alpha-1B	−1.834598101	D0-D7
CACNA2D1	voltage-dependent calcium channel alpha-2/delta-1	−5.013073193	D0-D7
CACNB3	voltage-dependent calcium channel beta-3	1.96153735	D0-D7
CACNG5	voltage-dependent calcium channel gamma-5	− 3.611690367	D0-D7
CACNG7	voltage-dependent calcium channel gamma-7	−2.217348209	D0-D7
CACNG8	voltage-dependent calcium channel gamma-8	−3.68477059	D0-D7
FGF2	fibroblast growth factor 2	−1.519463128	D0-D7
KITLG	KIT ligand	2.167299479	D0-D7
NTRK2	neurotrophic tyrosine kinase receptor type 2	8.559108654	D0-D7
KIT	proto-oncogene tyrosine-protein kinase Kit	2.471526791	D0-D7
FLT1	MS-like tyrosine kinase 1	−6.287270931	D0-D7
KDR	kinase insert domain protein receptor	−5.882467261	D0-D7
TGFB2	transforming growth factor beta-2	2.871123317	D0-D7
RASGRP2	RAS guanyl-releasing protein 2	−3.455335916	D0-D7
GNG12	guanine nucleotide-binding protein G(I)/G(S)/G(O) subunit gamma-12	−2.188972021	D0-D7
MYC	Myc proto-oncogene protein	−2.952930394	D0-D7
MECOM	ecotropic virus integration site 1 protein	3.315425714	D0-D7
PPM1B	protein phosphatase 1B	−1.591315588	D0-D7
ERBB4	receptor tyrosine-protein kinase erbB-4	4.531919812	D7-D13
CACNA1B	voltage-dependent calcium channel N type alpha-1B	1.88048051	D7-D13
CACNA2D3	voltage-dependent calcium channel alpha-2/delta-3	3.068907158	D7-D13
NTRK1	neurotrophic tyrosine kinase receptor type 1	4.469959879	D7-D13
TGFB2	transforming growth factor beta-2	2.803343326	D7-D13
KIT	proto-oncogene tyrosine-protein kinase Kit	−2.467841933	D13-D18
CACNG5	voltage-dependent calcium channel gamma-5	4.656489914	D13-D18
FGFR4	fibroblast growth factor receptor 4	−3.26316459	D13-D18
CACNA1E	voltage-dependent calcium channel R type alpha-1E	3.425325465	D18-D28
CACNG2	voltage-dependent calcium channel gamma-2	3.474133324	D18-D28
CACNG8	voltage-dependent calcium channel gamma-8	3.189789	D18-D28
KITLG	KIT ligand	2.028343818	D18-D28
EGFR	epidermal growth factor receptor	−2.084730311	D18-D28
FGFR1	fibroblast growth factor receptor 1	−2.081183963	D18-D28
FGFR2	fibroblast growth factor receptor 2	−3.256153332	D18-D28
FGFR3	fibroblast growth factor receptor 3	−5.009604609	D18-D28
KDR	kinase insert domain protein receptor	3.118215378	D18-D28
IL1RAP	interleukin 1 receptor accessory protein	3.502622162	D18-D28
PRKCB	classical protein kinase C beta type	1.642454101	D18-D28
PTPRR	receptor-type tyrosine-protein phosphatase R	2.837257876	D18-D28
PTPN5	tyrosine-protein phosphatase non-receptor type 5	2.472116702	D18-D28
MAPKAPK2	mitogen-activated protein kinase-activated protein kinase 2	−1.628879059	D18-D28
CDC25B	M-phase inducer phosphatase 2	−3.052843516	D18-D28
MAP3K5	mitogen-activated protein kinase kinase kinase 5	1.972512785	D18-D28

**Table 2:** DEGs identified in KEGG pathway: Calcium signaling. Differential expression indicates the average log_2_-fold change in RNA-Seq transcript levels from three independent experiments at each sampling period: D7 vs D0, D13 vs D7, D18 v D13, and D28 v D18. The *p*-values were ≤ 0.001. Negative log_2_-fold change corresponds to gene downregulation and the positive values indicate gene activation

Calcium signaling pathway			
Gene ID	Description	Differential expression	Time
HTR7	5-hydroxytryptamine receptor 7	−5.492892685	D0-D7
CACNA1B	voltage-dependent calcium channel N type alpha-1B	−1.834598101	D0-D7
CXCR4	C-X-C chemokine receptor type 4	4.604478969	D0-D7
ADCY2	adenylate cyclase 2	−3.460253861	D0-D7
STIM1	stromal interaction molecule 1	1.834422349	D0-D7
VDAC1	voltage-dependent anion channel protein 1	−1.32731076	D0-D7
GNA14	guanine nucleotide-binding protein subunit alpha-14	−5.014275635	D0-D7
TRDN	Triadin	−4.320486512	D0-D7
RYR2	ryanodine receptor 2	−4.07053211	D0-D7
RYR3	ryanodine receptor 3	1.71133494	D0-D7
ITPR2	inositol 1,4,5-triphosphate receptor type 2	−3.699823272	D0-D7
ITPR3	inositol 1,4,5-triphosphate receptor type 3	−2.406497059	D0-D7
ERBB4	receptor tyrosine-protein kinase erbB-4	4.531919812	D7-D13
CACNA1B	voltage-dependent calcium channel N type alpha-1B	1.88048051	D13-D18
CACNA1E	voltage-dependent calcium channel R type alpha-1E	3.425325465	D13-D18
GRIN2A	glutamate receptor ionotropic, NMDA 2A	2.854753149	D13-D18
P2RX3	P2X purinoceptor 3	2.552456853	D13-D18
CAMK4	calcium/calmodulin-dependent protein kinase IV	1.41910402	D13-D18
CHRM3	muscarinic acetylcholine receptor M3	3.978819163	D18-D28
HTR7	5-hydroxytryptamine receptor 7	3.808433488	D18-D28
GRIN1	glutamate receptor ionotropic, NMDA 1	3.726336735	D18-D28
GRIN2D	glutamate receptor ionotropic, NMDA 2D	2.087932817	D18-D28
CHRM2	muscarinic acetylcholine receptor M2	3.383752478	D18-D28
ADRA1A	adrenergic receptor alpha-1A	4.207285974	D18-D28
TACR3	tachykinin receptor 3	2.111829478	D18-D28
GRM1	metabotropic glutamate receptor 1	3.134222868	D18-D28
EGFR	epidermal growth factor receptor	−2.084730311	D18-D28
GNAL	guanine nucleotide-binding protein G(olf) subunit alpha	1.685044809	D18-D28
ADCY2	adenylate cyclase 2	3.240853085	D18-D28
STIM2	stromal interaction molecule 2	1.158068159	D18-D28
RYR2	ryanodine receptor 2	4.896050853	D18-D28
PRKCB	classical protein kinase C beta type	1.642454101	D18-D28

**Table 3:** DEGs identified in KEGG pathway: Neuroactive ligand-receptor interaction. Differential expression indicates the average log_2_-fold change in RNA-Seq transcript levels from three independent experiments at each sampling period: D7 vs D0, D13 vs D7, D18 v D13, and D28 v D18. The *p*-values were ≤ 0.001. Negative log_2_-fold change corresponds to gene downregulation and the positive values indicate gene activation

Neuroactive ligand-receptor interaction			
Gene ID	Description	Differential expression	Time
APLNR	apelin receptor	4.656030708	D0-D7
NMUR2	neuromedin U receptor 2	8.490690944	D0-D7
F2	coagulation factor II (thrombin)	2.589060962	D0-D7
CNR1	cannabinoid receptor 1	2.81817017	D0-D7
S1PR1	sphingosine 1-phosphate receptor 1	5.38497247	D0-D7
GRM4	metabotropic glutamate receptor 4	−3.861684955	D0-D7
GRIN2B	glutamate receptor ionotropic, NMDA 2B	−2.895136106	D0-D7
CHRNA3	nicotinic acetylcholine receptor alpha-3	2.7938378	D0-D7
HTR7	5-hydroxytryptamine receptor 7	−5.492892685	D0-D7
CHRNB4	nicotinic acetylcholine receptor beta-4	4.925250798	D0-D7
GRIA1	glutamate receptor 1	3.01762919	D0-D7
GRIA4	glutamate receptor 4	−2.647220311	D0-D7
GRIK1	glutamate receptor, ionotropic 42ainite 1	2.276415318	D0-D7
GRIK4	glutamate receptor, ionotropic 42ainite 4	−3.020412525	D0-D7
GRID2	glutamate receptor delta-2 subunit	−5.40793957	D0-D7
GLRA2	glycine receptor alpha-2	1.792820308	D0-D7
GLRB	glycine receptor beta	3.452103236	D0-D7
NR3C1	glucocorticoid receptor	−2.452514385	D0-D7
GHR	growth hormone receptor	2.976564346	D0-D7
CNR1	cannabinoid receptor 1	2.626395057	D7-D13
NR3C1	glucocorticoid receptor	3.341402421	D7-D13
GRIN2A	glutamate receptor ionotropic, NMDA 2A	2.854753149	D13-D18
CHRNA4	nicotinic acetylcholine receptor alpha-4	2.830329136	D13-D18
P2RX3	P2X purinoceptor 3	2.552456853	D13-D18
GRIA2	glutamate receptor 2	3.273675604	D13-D18
GRIA4	glutamate receptor 4	2.416455621	D13-D18
ADCYAP1R1	pituitary adenylate cyclase-activating polypeptide type I receptor	2.615605165	D13-D18
CHRM2	muscarinic acetylcholine receptor M2	3.383752478	D18-D28
CHRM3	muscarinic acetylcholine receptor M3	3.978819163	D18-D28
ADRA1A	adrenergic receptor alpha-1A	4.207285974	D18-D28
DRD2	dopamine receptor D2	−2.728065758	D18-D28
HTR7	5-hydroxytryptamine receptor 7	3.808433488	D18-D28
NMU	neuromedin U	−2.62455439	D18-D28
NPY5R	neuropeptide Y receptor type 5	3.804748523	D18-D28
HCRTR2	hypocretin (orexin) receptor 2	3.98239527	D18-D28
SSTR1	somatostatin receptor 1	6.807432954	D18-D28
TAC1	tachykinin 1	3.869735499	D18-D28
TACR3	tachykinin receptor 3	2.111829478	D18-D28
PTGER4	prostaglandin E receptor 4	2.824782168	D18-D28
GPR50	G protein-coupled receptor 50	4.747091061	D18-D28
LPAR1	lysophosphatidic acid receptor 1	1.500777066	D18-D28
S1PR1	sphingosine 1-phosphate receptor 1	2.506524278	D18-D28
ADCYAP1R1	pituitary adenylate cyclase-activating polypeptide type I receptor	−2.231972171	D18-D28
GRM1	metabotropic glutamate receptor 1	3.134222868	D18-D28
GRIN1	glutamate receptor ionotropic, NMDA 1	3.726336735	D18-D28
GRIN2D	glutamate receptor ionotropic, NMDA 2D	2.087932817	D18-D28
GRIN3A	glutamate receptor ionotropic, NMDA 3A	2.914543821	D18-D28
CHRNA3	nicotinic acetylcholine receptor alpha-3	−2.670435709	D18-D28
GRIA2	glutamate receptor 2	2.698334339	D18-D28
GRIA4	glutamate receptor 4	1.923861676	D18-D28
GRID2	glutamate receptor delta-2 subunit	4.217720694	D18-D28
GRIN2B	glutamate receptor ionotropic, NMDA 2B	4.109294839	D18-D28
GLRA3	glycine receptor alpha-3	5.577349539	D18-D28
GLRB	glycine receptor beta	1.392250382	D18-D28

**Table 4:** DEGs identified in KEGG pathway: Axon guidance. Differential expression indicates the average log_2_-fold change in RNA-Seq transcript levels from three independent experiments at each sampling period: D7 vs D0, D13 vs D7, D18 v D13, and D28 v D18. The *p*-values were ≤ 0.001. Negative log_2_-fold change corresponds to gene downregulation and the positive values indicate gene activation

Axon guidance			
Gene ID	Description	Differential expression	Time
NTNG1	netrin-G1	−2.536297436	D0-D7
DCC	deleted in colorectal carcinoma	2.502983549	D0-D7
NTN1	netrin 1	3.198672486	D0-D7
FYN	tyrosine-protein kinase Fyn	1.772436052	D0-D7
RGMA	repulsive guidance molecule A	3.097407276	D0-D7
CXCR4	C-X-C chemokine receptor type 4	4.604478969	D0-D7
CXCL12	C-X-C motif chemokine 12	−4.220104913	D0-D7
EPHA3	Eph receptor A3	3.43500774	D0-D7
EPHA4	Eph receptor A4	1.860959561	D0-D7
EPHA7	EphA7	2.088277014	D0-D7
EPHB2	Eph receptor B2	1.873701283	D0-D7
EPHB3	Eph receptor B3	5.416063376	D0-D7
ENAH	Enabled	−0.89497636	D0-D7
SLIT1	slit 1	5.447826267	D0-D7
ROBO2	roundabout, axon guidance receptor 2	−2.472827128	D0-D7
NRP1	neuropilin 1	4.117109594	D0-D7
DPYSL5	dihydropyrimidinase-like 5	4.921620042	D0-D7
PTCH1	patched 1	3.517640353	D0-D7
BOC	brother of CDO	4.811514207	D0-D7
BMP7	bone morphogenetic protein 7	3.232399315	D0-D7
BMPR1B	bone morphogenetic protein receptor type-1B	1.78373296	D0-D7
DCC	deleted in colorectal carcinoma	2.626762708	D7-D13
EPHA3	Eph receptor A3	2.836614823	D7-D13
PAK3	p21-activated kinase 3	1.64575883	D13-D18
NTNG1	netrin-G1	2.991377261	D13-D18
EPHA5	Eph receptor A5	3.15278798	D13-D18
SLIT1	slit 1	2.183813571	D13-D18
SLIT2	slit 2	2.342825102	D13-D18
NTNG1	netrin-G1	2.430765649	D18-D28
TRPC5	transient receptor potential cation channel subfamily C member 5	2.464006156	D18-D28
RGS3	regulator of G-protein signaling 3	−1.814236062	D18-D28
CXCL12	C-X-C motif chemokine 12	3.751564688	D18-D28
EPHA5	Eph receptor A5	3.524159164	D18-D28
EPHA6	Eph receptor A6	2.585950072	D18-D28
BOC	brother of CDO	−1.858286661	D18-D28
WNT4	wingless-type MMTV integration site family, member 4	2.873234122	D18-D28

**Table 5:** DEGs identified in KEGG pathway: Cholinergic synapse. Differential expression indicates the average log_2_-fold change in RNA-Seq transcript levels from three independent experiments at each sampling period: D7 vs D0, D13 vs D7, D18 v D13, and D28 v D18. The *p*-values were ≤ 0.001. Negative log_2_-fold change corresponds to gene downregulation and the positive values indicate gene activation

Cholinergic synapse			
Gene ID	Description	Differential expression	Time
GNG12	guanine nucleotide-binding protein G(I)/G(S)/G(O) subunit gamma-12	−2.18897	D0-D7
CACNA1B	voltage-dependent calcium channel N type alpha-1B	−1.8346	D0-D7
KCNQ3	potassium voltage-gated channel KQT-like subfamily member 3	−2.96129	D0-D7
KCNJ12	potassium inwardly-rectifying channel subfamily J member 12	3.350697	D0-D7
CHRNA3	nicotinic acetylcholine receptor alpha-3	2.793838	D0-D7
CHRNB4	nicotinic acetylcholine receptor beta-4	4.925251	D0-D7
ITPR2	inositol 1,4,5-triphosphate receptor type 2	−3.69982	D0-D7
ITPR3	inositol 1,4,5-triphosphate receptor type 3	−2.4065	D0-D7
ADCY2	adenylate cyclase 2	−3.4602539	D0-D7
ADCY6	adenylate cyclase 6	1.892735	D0-D7
CREB5	cyclic AMP-responsive element-binding protein 5	2.033579	D0-D7
FYN	tyrosine-protein kinase Fyn	1.772436	D0-D7
CAMK4	calcium/calmodulin-dependent protein kinase IV	1.419104	D7-D13
ChAT	choline O-acetyltransferase	4.392642	D13-D18
CACNA1B	voltage-dependent calcium channel N type alpha-1B	1.880481	D13-D18
SLC5A7	solute carrier family 5 (high affinity choline transporter), member 7	4.94003258	D13-D18
KCNQ2	potassium voltage-gated channel KQT-like subfamily member 2	3.013059	D13-D18
CHRNA4	nicotinic acetylcholine receptor alpha-4	2.830329	D13-D18
CHRM3	muscarinic acetylcholine receptor M3	3.97881916	D18-D28
ChAT	choline O-acetyltransferase	1.799457	D18-D28
ACHE	Acetylcholinesterase	2.982588	D18-D28
CHRM2	muscarinic acetylcholine receptor M2	3.383752	D18-D28
SLC5A7	solute carrier family 5 (high affinity choline transporter), member 7	2.363264	D18-D28
KCNQ3	potassium voltage-gated channel KQT-like subfamily member 3	3.701006	D18-D28
CHRNA3	nicotinic acetylcholine receptor alpha-3	−2.67044	D18-D28
PRKCB	classical protein kinase C beta type	1.642454	D18-D28
ADCY2	adenylate cyclase 2	3.240853	D18-D28

### MAPK-signaling pathway

MAPK-signaling (Table 1) has an important role in mediating neuronal differentiation and survival [23]. Consistent with our expectations, the transition from iPSCs to NSCs was guided by inhibition of signal transduction pathways supporting self-renewal and activation of genes regulating neuron-specific development pathways. Gene expression of Neurotrophic Tyrosine Kinase Receptor type 2, *NTRK2*, was prominently upregulated at D7 when the NSC population was established, while a subset of genes encoding for γ-subunits of voltage-dependent calcium-channels (Cavs) were dramatically downregulated (*CACNG5*, *CACNG7*, and *CACNG8*), along with *MYC*, Ras guanyl-releasing protein 2 (*RASGRP2*), and *GNG12*. Gene groups encoding the auxiliary subunits of high-voltage activated (HVA) Cavs were upregulated during the early stages of neuronal development [24]: *CACNB3* (induced 4-fold at D7) and *CACNA2D3* (16-fold activation at D7-D13). The late stage of MN formation (D18-D28) was marked by upregulation of *CACNA1E* and *CACNG2*. Several gene groups encoding auxiliary subunits of low-voltage activated (LVA) Cavs (*CACNG5* and *CACNG8*), were initially downregulated in NSC and later upregulated during the MN maturation stage (D18-D28). Accumulation of Cavs gene transcripts throughout the MN differentiation process is consistent with the increased excitability of neuronal cells (Fig. 2).

Other key components of MAPK-signaling showed oscillatory patterns of gene expression throughout MN formation and maturation. Gene transcripts of *KIT* and its ligand, *KITLG*, which function in stem cell maintenance, were upregulated in NSCs (D7) and downregulated during the transition from MNP to eMN (D13-D18). *KITLG* transcription was again upregulated in MNs (D18–28). KIT signaling promotes cell survival through the activation of PIK3, PLC, and AKT1 pathways and we detected upregulation of *KITLG* gene expression in MNs which may be a response to the addition of IGF-1 in the culture media. Upregulation of various genes regulating cell survival and proliferation in the post-mitotic MN, such as *KDR*, *PTPRR*, *PTPN5*, and *PRKCB*, further exemplifies the MN response to IGF-1 pro-survival stimulus.

### Calcium (Ca^2+^) signaling pathway

Ca^2+^-signaling (Table 2) is a critical component of synaptic activity and neuronal function [25]. Our RNA-Seq data indicated the conversion of iPSCs into NSCs is marked by a significant downregulation of a gene encoding for a serotonin-specific GPCR, 5-hydroxytryptamine receptor 7 (*HTR7*). In addition, adenylate cyclase 2 (*ADCY2*) was also downregulated at this time and is known to act immediately downstream of HTRs. Together, the downregulation of *HTR7* and *ADCY2* indicates a functional silencing of the cAMP-dependent signaling pathway. Furthermore, multiple genes involved in the activation of Ca^*2+*^ signaling were downregulated: GPCR subunit alpha-14 (*GNA14*), an activator of phospholipase C (LPC); two gene groups encoding inositol 1,4,5-triphosphate receptors (*ITPR2* and *ITPR3*); and ryanodine receptor 2 (*RYR2*). (Fig. 5d and Additional File 2) One GPCR from the chemokine family, *CXCR4*, involved in the cytosolic Ca^*2+*^ mobilization and cell migration, was also markedly upregulated (16-fold) during the transition into NSCs.

A hallmark of the NSC to MNP transition was the accumulation of receptor tyrosine-protein kinase erB-4 (*ERBB4*) transcripts. ERBB4 is activated by epidermal growth factor proteins, neuregulins2/3, to shape the development of neuronal cells through activation of MAPK3/ERK [26, 27]. *ERBB4* expression was upregulated by 18-fold in D7-D13, followed by upregulation of *CACNA1B*, *CACNA1E*, *GRIN2A* and *P2RX3* (D13-D18). Formation of eMNs (D13-D18) coincided with upregulated gene expression of HVA N- and R-type (*CACNA1B* and *CACNA1E*) voltage-dependent Ca^*2+*^ channels, glutamate ionotropic receptor (*GRIN2A*), and the ligand-gated ion channel responsible for peripheral pain responses, *P2RX3*. These gene expression profiles indicate the selective pressure of chemical stimuli lead to the formation of eMNs with characteristics of the sympathetic nerve system [28].

In the final stage of MN maturation (D18-D28), we observed upregulation of gene groups activating Ca^*2+*^ signaling through the cAMP-dependent pathway: *HTR7* and *ADCY2*. We also detected upregulated expression of the intracellular Ca^*2+*^ ryanodine receptor 2 (*RYR2*), signifying cell readiness to release Ca^*2+*^ from the sarcoplasmic reticulum in response to adrenergic (sympathetic) stimulation (Fig. 5d). This finding was consistent with the upregulation of *ADRA1A*, which encodes the adrenergic receptor alpha subunit 1α. Furthermore, gene activation of both ionotropic (*GRIN1* and *GRIN2D*) and metabotropic (*GRM1*) glutamate receptors, together with the upregulation of neuromedin-K receptor (*TACR3*), accentuated the role of the IP3-Ca^*2+*^ second messenger system to activate ERK1/2 as well as classical PKC signaling pathways [29–31]. We also detected higher levels of *PRKCB* gene transcripts in MNs compared to eMNs (Table 2).

Interestingly, G-coupled muscarinic acetylcholine receptors (mAChRs) were upregulated in mature MNs (D28). Genes encoding for excitatory (*CHRM3*) and inhibitory (*CHRM2*) mAChRs were specifically activated in late stage MN maturation (Table 2, D18-D28). The excitatory mAChRs stimulate PLC, triggering IP3 and diacylglycerol signaling pathways (Additional file 2). Since mAChRs are present on parasympathetic neurons [32], our data suggest that the iPSC-induced MNs bear the excitatory characteristics of parasympathetic neurons.

### Neuroactive ligand-receptor interactions

The majority of neuronal receptors are GPCRs and they function to regulate signal transduction pathways and shape cellular physiological responses. Our genome wide transcriptome analysis revealed that iPSC-induced MNs differentiation is marked by the expression of neuroactive receptors which are responsive to glutamate, ACh, and catecholamines, orexin, and prostaglandins (Table 3).

During the first step of iPSC commitment to a neuronal lineage, multiple glutamate receptors were altered, notably, gene expression levels of two ionotropic glutamate receptors were upregulated in the NSCs, *GRIA1* and *GRIK1*, while their paralog genes, *GRIA4* and *GRIK4*, were downregulated. This suggests a specific requirement for type 1 ionotropic glutamate receptors early in neuronal development. Neuromedin and apelin receptors (*NMUR2* and *APLNR*) were upregulated in NSCs, as well as GPCR response to sphingosine-1-phosphate signaling receptor (*S1PR1*), suggesting changes in cytoskeleton dynamics and mitosis [33]. Genes encoding for nicotinic acetylcholine receptors (nAChR) and cannabinoid receptors (*CNR1*and *CHRNB4*) were upregulated during the conversion of NSCs into MNPs and remained activated until eMNs were formed.

Formation of eMNs was marked by reactivation of genes encoding glutamate receptors which were downregulated at the NSC stage. Gene activity of various ionotropic glutamate receptors peaked when eMNs became mature MNs (D18-D28). Activation of genes regulating the synthesis of various neurotransmitters defined the transition from eMNs to MNs. The transcript levels of the neuropeptide *tachykinin*, and its receptor (*TACR3*), were upregulated in MNs. In spinal neurons, tachykinins evoke synthesis and release of ACh, histamine, catecholamines, and GABA. We also detected differential expression of the somatostatin receptor (*SSTR1*) indicating that the mature MN population can secrete neurotransmitters [34]. The glucocorticoid receptor (*NR3C1*) was upregulated in MNPs and MNs and two new classes of neuroactive receptors were upregulated in the last stage of MN maturation: hypocretin/orexin (*HCRTR2*) and prostaglandin E receptor 4 (*PTGER4*). Our transcriptomic data indicate that the replacement of ionotropic nAChR (*CHRNA3*) and dopamine receptor (*DRD2*) expression with adrenergic receptor (*ADRA1A*) and mAChRs (*CHRM2* and *CHRM3*) marked the formation of mature MNs. The controversial activation of mAChRs and genes encoding excitatory neurotransmitters, including glutamate and catecholamines (ADRA1A), could stem from the formation of a heterogeneous population consisting of parasympathetic and sympathetic MNs.

### Axon guidance and cholinergic synapse

Pathways regulating axon guidance and synapse development were upregulated during iPSC-to-MN differentiation (Tables 4 and 5, respectively). These functions are critical for neuronal development, maintenance, and repair mechanisms.

We found netrin-G1 (*NTNG1*) to be initially downregulated as iPSCs transitioned into NSC, but then upregulated in eMNs and MNs from D13–28. Deleted in colorectal carcinoma *(DCC*) was also significantly upregulated as iPSCs transition through NSCs to become MNPs, indicating its critical role in prompting axon extension. *SLIT1* was upregulated at D7 and both *SLIT1* and *SLIT2* were upregulated as the NSCs became MNPs (D7-D13). HB9 tightly regulates Robo2 expression to regulate motor axon guidance in ventrally-projecting MNs [35] and we observed *ROBO2* downregulation in NSCs, during which time HB9 transcripts were initially accumulating (Fig. 6g).

In vivo as MN axons extend, they have the potential to terminate on a muscle fiber at a cholinergic synapse, the site of ACh neurotransmission. In the pre-synaptic neuron, ACh synthesis is driven by choline O-acetyltransferase (ChAT) and we observed *ChAT* gene expression to be increased in both eMNs and MNs. Expression of ion-channel-coupled nAChR subunits were also significantly altered: *CHRNA3* was regulated in NSCs but downregulated in MNs and *CHRNA4* was upregulated at the eMN stage. In the postsynaptic membrane these receptor components would contribute to neuroactive ligand-receptor interactions as previously described. Gene expression of acetylcholinesterase (*AChE*) was significantly increased late in MNs maturation (D18-D28), which encodes an enzyme responsible for the hydrolysis of ACh into choline and acetic acid, a critical step in ACh recycling in the cholinergic synapse. Membrane transporter solute carrier family 5 member 7 (SLC5A7) is then able to import choline back into a cholinergic neuron for subsequent ACh synthesis. We found gene expression of *SLC5A7* to be increased in both the eMNs and mature MNs.

### Heterogeneity of MN populations

As previously reported [8], in vitro differentiation of human iPSC resulted in heterogeneous populations consisting of several MN subtypes. Applying the same differentiation protocol [6] and quantitative image analysis (Fig. 1d-e), we observed that two pan-MN transcription regulators ISL-1and HB9 were present in ~ 60 (±12%) and ~ 85 (±15%) of the cell populations, respectively (Fig. 1d-e). Thus, our quantitative image analysis data is in agreement with the 68 ± 7% double positive ISL1^+^/HB9^+^ MN populations reported by Thiry et al [8]. Based on our image analysis on single cell level (Fig. 1) and quantitative RT-PCR of bulk transcriptome (Fig. 6) we could infer that iPSC-derived neuronal populations are highly enriched on MN in general although without detailed analysis of the molecular markers characteristic to each MN subtype we are not able to conclude which one is predominant. Combination of transcription master regulators' activity determines the MN functional specifications (Table 6). Based on the quantitative image analysis, majority of the MNs were positive for the HB9 gene (Fig. 1c-d), which remains activated in mature MNs from the median and hypaxial motor columns innervating the long muscles of the back and the respiratory muscles, respectively, while HB9 is downregulated in the medial neurons of the lateral motor column (LMC) innervating the limb muscles. The meta-transcriptome data from our study showed significant (*p* < 1e-5) activation (log_2_ > 2) of key molecular markers specific to various MN subtypes ranging from the preganglionic column (SMAD1 and ZEB2), spinal accessory column (RUNX1 and PHOX2B), and the lateral LMC (FOXP2 and ALDH1A2). Taken together, our results (summarized in Table 6) strongly indicate that in vitro derived MN populations are heterogeneous consisting of several functional subtypes.

**Table 6:** MN specification based on log_2_-fold change in RNA-Seq transcript levels at D28 versus D0 of the differentiation protocol. The *p*-values were calculated with the R-package. Negative log_2_-fold change corresponds to gene downregulation and the positive values indicate gene activation. *↓* indicates the gene is expected to be downregulated in the MN subtype

	Gene Biomarkers	Fold change	***p***-value
**General motor neuron**	*HB9*	4.29740102	0.03478379
	*ISL1*	9.38120075	1.39E-11
	*ISL2*	−2.71940491	0.12115956
	*LHX3*	5.98518477	1.09E-05
**Hypaxial motor column**	*ETV1*	−4.14432026	8.33E-05
	*HB9*	4.29740102	0.03478379
	*ISL1*	9.38120075	1.39E-11
	*ISL2*	−2.71940491	0.12115956
**Pre-ganglionic column**	*SMAD1*	1.61250783	3.71E-10
	*NOS1*	−1.1688341	0.1409026
	*ZEB2*	4.94457944	9.71E-19
	*FOXP1*	0.76366376	0.10008361
**Spinal accessory column**	*ALCAM*	0.82274029	0.14709124
	*ISL1*	9.38120075	1.39E-11
	*RUNX1*	4.90888056	1.01E-20
	*PHOX2B*	9.18779756	1.30E-06
**Medial LMC**	*↓ HB9*	4.29740102	0.03478379
	*ISL2*	−2.71940491	0.12115956
	*FOXP2*	5.83531355	9.82E-56
	*ALDH1A2*	5.96267067	8.55E-13
**Lateral LMC**	*↓ ISL1*	9.38120075	1.39E-11
	*ISL2*	−2.71940491	0.12115956
	*FOXP2*	5.83531355	9.82E-56
	*ALDH1A2*	5.96267067	8.55E-13
**Median motor column**	*HB9*	4.29740102	0.03478379
	*ISL1*	9.38120075	1.39E-11
	*ISL2*	−2.71940491	0.12115956
	*LHX3*	5.98518477	1.09E-05

## Discussion

This comprehensive study documents transcriptomic and morphological changes of iPSCs as they differentiate into motor neurons (MNs) in vitro. We performed RNA-Seq meta-transcriptome analysis of human iPSC and the four cell types representing stages of spinal motor neuron differentiation (NSC, MNP, and eMN) and maturation (MN). The results from our genome-wide transcriptome study revealed basic developmental principles of in vitro neurogenesis from iPSC that have not been elucidated by previous studies while confirming the regulatory role of TGFβ, Notch, and Shh signaling pathways in the formation of adult spinal motor neurons. We further corroborated the findings from the next-generation RNA-Seq analysis with RT-qPCR gene expression assays and immunohistochemistry profiles of key pluripotency and MN markers. Applying novel OPaver software we found strict temporal correlation between the formation of functional neuronal network connections on a MEA chip and the expression of genes involved in the regulation of Ca^2+^ signaling. We found that cAMP-regulated Ca^2+^ signaling was inhibited on gene transcript level when iPSC traversed through the neurodevelopmental stages and was reactivated in the final stage (D28) of MN maturation when we were able to detect neuronal synaptic activity via recordings of spontaneous AP firings.

### Inhibition of Ca^2+^ signaling is required for the transition from iPSC to NSC

Intracellular Ca^*2+*^ levels, known as calcium transients, regulate neuronal subtype and neurotransmitter specifications [36, 37]. Our global transcriptome data showed activation of genes regulating Ca^*2+*^ entry, specifically voltage-sensitive calcium channels and ionotropic glutamate receptors, not sooner than at the eMN developmental stage (D18 vs D13, Table 1 and Fig. 5d). Ca^*2+*^ signaling comprises a cascade of molecular interactions and biophysical events, which translate extracellular signals to intracellular responses via increase of cytoplasmic Ca^*2+*^. This can be activated by neurotransmitters, hormones, and growth factors, chemical and electrical stimuli, causing membrane excitation. Two fundamental mechanisms regulate Ca^*2+*^entry through protein channels: voltage-dependent Ca^*2+*^ channels and ligand-gated channels. The latter are highly diverse, non-Ca^*2+*^-specific, and greatly represented by the family of guanine-binding GPCR [38]. Significant upregulation in differentiated MN of high-voltage activated Cavs genes, such as *CACNB3*, is responsible for tight control on intracellular Ca^*2+*^ signaling through regulation of calcium (Ca^*2+*^) entry and direct interaction with phospholipase C-coupled (PLC) and inositol trisphosphate (IP3) receptors [39]. Upregulation of *CXCR4* by 16-fold at D0-D7 underlines the importance of intracellular Ca^*2+*^ signaling inhibition as the neuronal cell lineage commitment process begins. The CXCR4/CXCR7 complex recruits β-arrestin to trigger the canonical GPCR pathway activating ERK1/2, p38, and SAPK, while inhibiting both Ca^*2+*^ mobilization and cAMP signaling [40]. Upregulation of neuromedin and apelin receptors (*NMUR2* and *APLNR*) in NSCs suggest a role for phosphoinositide (PI) signaling pathways that inhibit cAMP production upon Ca^*2+*^ mobilization and possibly in regulating cytoskeleton dynamics, cell growth, and hormone release [41, 42].

Our findings underline the role of temporal gene regulation of Ca^2+^ signaling in motor neuron development in vitro. It has been previously demonstrated that voltage-gated Ca^*2+*^ influx activated by cAMP is instrumental in the maturation of neuronal progenitor cells into functional neurons [43]. The comparative transcriptome study presented here revealed that genes regulating cAMP synthesis (ADCY2), voltage-gated calcium channels (CACNA1B and CACNG5), and the receptor regulating the intracellular Ca^2+^ homeostasis (RYR2) were immediately inhibited upon transition of iPSC into NSC and were re-activated in mature MN. Collectively, our data suggests that inhibition of pathways regulating the Ca^2+^ transients is required for the successful transition from pluripotency to neuronal progenitor cells. Our findings corroborate previous studies that have demonstrated that low cellular excitability is vital for cell migration while increase in the Ca^*2+*^ transients stops migration and promotes dendrite formation in cortical neurons [44].

### MN specification in vitro is driven by the chemical stimuli in growth media and bare the characteristics of the parasympathetic nervous system

The transcriptome analysis revealed the underlying molecular changes in differentiation of iPSC to mature MNs. Specifically, we observed that short-term inhibition of TGFβ signaling was sufficient to push iPSCs into NSCs and that continuous activation of Notch and Shh signaling pathways ensured morphogenesis and cell survival throughout the MN differentiation and maturation process. While the majority of changes were characteristic of neuronal development, deeper analysis revealed the involvement of more unexpected genes as well as unique temporal changes. For example, the oscillatory pattern of *SKIL* gene expression throughout the neuronal cell differentiation process suggests a regulatory feed-back loop that balances survival and cell differentiation programs. *PTCH1*, a Shh receptor gene, is consistently and significantly upregulated from D7 to D28 suggesting a role as a positive regulator in neuronal cell division. Binding of Shh to PTCH1 results in the release of the smoothened protein initiating cell proliferation. Upon ligand binding, PTCH1 is trafficked away from the Shh positive regulator, G-coupled receptor SMO, resulting in downstream signal transduction [45]. Downregulation of *PTCH1* by D28 supports the possible inhibition of cell proliferation and subsequent differentiation to neurons. *ARHGAP36* and *CRMP1*, both of which are downregulated simultaneously, encode for Rho GTPase activating protein 36 and collapsin response mediator protein 1, are implicated in semaphorin-induced growth cone and axon guidance [46, 47] and may regulate cell division and morphogenesis of MNs.

Applying quantitative gene transcript analysis and immunohistochemistry, we observed that *ChAT*, a marker of mature neurons, was upregulated as soon as iPSC committed to a neuronal lineage (D7). In addition, the well-orchestrated expression of key genes such as *Nestin*, *Pax6*, *NgN2*, *MAP2*, *Isl1* and *HB9* corroborated the phenotypic changes to MNs. The patterns of gene expression reported here are consistent with similar studies using small molecules to drive neuron reprogramming from astrocytes [3] or fibroblasts [48].

While Shh drives motor neuron formation over intermediate neurons, there are multiple subtypes of MNs. In the final stage of MN maturation, we observed that genes encoding ionotropic nAChR (*CHRNA3*) and dopamine receptor (*DRD2*) were downregulated while adrenergic receptor (*ADRA1A*) and mAChRs (*CHRM2* and *CHRM3*) gene expression was activated. Since mAChRs are found exclusively on the neurons from the parasympathetic system [49] our data indicates that in vitro iPSC-derived MN in monoculture have the characteristics of the parasympathetic neurons of the peripheral nervous system.

In addition to the parasympathetic neuron-specific mAChRs gene activation, genes encoding excitatory neurotransmitters specific to the sympathetic neurons, including glutamate and catecholamines (ADRA1A), were significantly upregulated in the population of mature motor neurons. This could stem from the formation of a heterogeneous population consisting of parasympathetic and sympathetic MNs. Previous studies based on single-cell RNA-Seq analysis of iPSC-derived spinal MN have demonstrated that the protocol for in vitro differentiation produces a mixed population of MN subtypes with a predominant (58%) sub-population of lateral MNs and several minority sub-populations, including hypaxial motor column (19%) and median motor column (6%) MNs [8].

### The neuroactive ligands define electrophysiological activity of in vitro iPSC-derived mature MNs

The electrophysiological activity of mature MN reflects their ability to form functional neuronal network connections. Neuronal precursors and neurons are capable of spontaneous electrical activity [50], and we observed increased electrical activity as synapses and neural circuits formed. As iPSCs differentiated after plating on the MEAs, dendrite projections formed as axons stretched between neuronal cell bodies. These networks of MNs created numerous synapses to propagate nerve impulses. The MEAs were able to capture spontaneous firings of extracellular action potentials as single spikes of activity early in differentiation which progressed to more frequent firing bursts as the iPSC-derived MNs matured, demonstrating peak activity on D31. While the length and amplitude of an action potential are always the same, an increase in the stimulus caused an increase in the frequency of an action potential indicative of an enhanced response.

MNs are often characterized by their role in forming cholinergic synapses at NMJs. MN axons terminate on muscle fibers and nerve impulses are translated into muscle contractions as the neurotransmitter ACh is released from the presynaptic MN terminal for uptake by postsynaptic ACh receptors on the target muscle cell [1]. Understanding the underlying mechanism of cholinergic development provides insights about potential treatments for neurodegenerative diseases and strategies to develop countermeasures to chemical toxicology exposure. AChE inhibitors are used to treat Alzheimer’s and Parkinson’s diseases [51, 52] and cholinergic agonist treatments have shown to improve memory function [53]. Similar strategies have been employed to treat organophosphate-induced neurotoxicity [54, 55]. Here we show how culture conditions imprint distinct fates and future efforts to co-culture of MNs with other cell types, such as muscle, may cause different neuronal specification. Surprisingly, we observed the expression of nAChRs typically found on the NMJ to be downregulated in mature MN monocultures while the mAChRs were upregulated. These results can further be applied to characterize aberrant neuronal functions following neurodegeneration or exposure to chemical toxins.

## Conclusions

This study revealed significant changes to the transcriptome, protein expression, and electrical function as iPSCs differentiated into mature MNs. To ensure cellular mobility that is essential for tissue layer formation, genes regulating Ca^2+^ signaling are downregulated throughout the cell differentiation process and are activated in mature MN. Differentiation of iPSC into MN in vitro monocultures leads to formation of both parasympathetic and sympathetic neuronal types. Downregulation of tumor suppressing genes upon chemical conversion of iPSC to mature MN underlines the danger from direct application of the technology in vivo. Understanding the underlying molecular and cellular cues involved in MN differentiation of iPSCs has the potential to enable the discovery of novel treatments for neural injuries.

## Methods

### Culturing and differentiating iPSCs

Human iPSCs (WTC-11, Coriell Institute) were cultured and maintained on vitronectin (ThermoFisher Scientific) treated culture plates in Essential8 medium (ThermoFisher Scientific). Differentiation of iPSCs into MNs was directed as previously described [6] with slight modifications that substituted the NSC growth in suspension (D7-D13) with a growth on vitronectin substrate to improve the cell survival rate and to ensure gene expression profile characteristic to motor neuron progenitor cells (MNP). Briefly, iPSCs were cultured in neural media which consisted of 1:1 DMEM/F12 and Neurobasal medium supplemented with N2, B27, 1x Glutamax and 1x penicillin/streptomycin (all from ThermoFisher Scientific), and 0.1 mM ascorbic acid (StemCell Technology). On days 0–6 3 μM CHIR99021 (StemCell Technology), 2 μM DMH-1 (Tocris) and 2 μM SB431542 (StemCell Technology) were added to the neural medium; days 6–12 the same media was used with the addition of 0.1 μM RA (StemCell Technology) and 0.5 μM Pur (Sigma); days 12–18 cells were maintained with 0.1 μM RA and 0.5 μM Pur added to the neural media; finally from day 18 on cells were cultured with 0.5 μM RA, 0.1 μM Pur and 0.1 μM CpdE (StemCell Technology), IGF-1, BDNF, and CNTF (all from R&D Systems, 10 ng/ml each). For optimum neuronal network formation, cells at the MNP differentiation stage (D12) were plated on MEA at 60% area density.

### RNA extraction

Cells were lifted with accutase, pelleted by centrifugation, and stored at − 20 °C. Total RNA was extracted from cell pellets using RNeasy Mini Kit (Qiagen), following the recommendations of the manufacturer. After DNase digestion by Turbo DNA-free kit (ThermoFisher Scientific), samples were quantified and divided for qPCR and transcriptomic analyses.

### RNA sequencing analysis

Extracted and DNase-treated RNA was quantified using the Qubit 4 Fluorometer (ThermoFisher) with the High Sensitivity RNA reagents and Bioanalyzer (Agilent) with RNA 6000 Pico reagents. Ribosomal depletion, DNA conversion, and library preparation was performed on all samples using the Illumina TruSeq Stranded Total RNA kit. 151 base pair reads were sequenced on the Illumina NextSeq. Across fifteen samples (three independent experiments x five time points) the total number of reads generated for each sample ranged from approximately 26 million to 40 million reads. Sequencing data was quality trimmed using FaQCs [56] with a quality score cutoff of Q20. Differential expression analysis was performed using PiReT [57] V 0.3.2 and utilizing DEseq2 [58] default parameters and setting a q-value of 0.05 (false discovery rate metric). The experimental design file (provided in the supplementary material) was used to dictate the replicate sample ID’s and sequencing data to be used in the PiReT analysis. Human genome version hg38 was used as the reference genome. KEGG [59, 60] pathway mapping was performed using Omics Pathway Viewer - ‘OPaver’ (Li, unpublished). Raw RNA-Seq reads were deposited in the NCBI SRA database under the accession numbers SRR11994167- SRR11994181. Metadata for each sample are also accessible under NCBI BioProject PRJNA638768.

### Quantitative reverse transcription PCR (RT-qPCR)

Three independent experiments were run in duplicate using a 7500 Fast Real-Time PCR System (Applied Biosciences). Fifty ng of each RNA sample were probed for motor neuron differentiation markers using Taqman RNA-to-CT 1-Step Kit (Applied Bioscience) in a 25 μl volume according to the manufacturer’s instructions. Taqman probes included NEUROG2 (Hs00935087_g1), ChAT (Hs00758143_m1), Isl1 (Hs01099686_m1), PAX6 (Hs01088114_m1), MAP2 (Hs00258900_m1), Nestin (Hs04187831_g1), Oct4 (Hs00999632_g1), and HB9 (Hs00907365_m1). Two endogenous controls, actin (Hs99999903_m1) and GAPDH (Hs01922876_u1), were analyzed by RT-qPCR and no significant differences were observed in their expression levels (data not shown); thus, all data shown are normalized to GAPDH.

### Immunocytochemistry staining and analysis

Nunc Lab-Tek chamber slides (ThermoFisher Scientific) were coated with vitronectin, seeded, and subsequently fixed with 4% paraformaldehyde, permeabilized with 0.4% Triton X-100, blocked with 3% BSA in PBS for at least 1 h. Samples were incubated overnight at 4 °C with primary antibody solutions (Table 6) diluted in PBS containing Image-iT FX signal enhancer. Cells were washed with PBS three times prior to incubation with NucBlue Fixed Cell Reagent, Image-iT FX signal enhancer, and secondary Alexa-488-, Alexa 555-, or Alexa 647-conjugated antibodies at 37 °C for 2 h (1:1000 dilution, ThermoFisher Scientific and Jackson ImmunoResearch). Table 7 summarizes the antibodies and their concentrations applied in this study. After three PBS washes, the media chambers were removed from the glass slide, coverslips were mounted using ProLong Diamond Antifade Mountant and cells were examined using fluorescence microscopy (Zeiss Observer Z.1). For biomarker quantification, images were acquired with an AxioCam camera connected to Axio Observer Z1 microscope, using ZEN software. For each culture, images were taken with a 20X objective choosing fields with > 100 cells. Images from 5 random fields per culture condition were analyzed with Image J with the following parameters: (i) manual threshold, (ii) fill holes, (iii) enhance contrast and saturated pixels at 0.3%, (iv) watershed separation, and (v) particle analysis at size 100-infinity and circularity at 0.2–0.1. The fraction of cells expressing biomarker proteins was calculated as percent of total cells labeled with DAPI.

**Table 7:** List of primary antibodies

Antibody	Species	Dilution	Source
Oct4	Rabbit	1:200	Cell Signaling Technology (Cat# 2750S)
Nestin	Rabbit	1:200	Abcam (Cat# ab105389)
Pax6	Mouse	1:50	Developmental Studies Hybridoma Bank (Cat# Pax6)
MAP2	Mouse	1:200	ThermoFisher (Cat# MA5–12823)
HB9 / MNX1	Mouse	1:50	Developmental Studies Hybridoma Bank (Cat# 81.5C10)
ChAT	Goat	1:100	Milipore (Cat#AB144P)
beta-III-tubulin	Rabbit	1:1000	Abcam (Cat# ab18207)
Synaptophysin [SY38]	Mouse	1:100	Abcam (Cat# ab8049)

### Functional analysis of MNs on microelectrode array (MEA)

Cells were seeded on MEA chips, either in 60MEA200/30iR-Ti arrays or 24-well Plate with PEDOT Electrodes on Glass, 24 W300/30G-288 (Multichannel Systems). MEA’s were coated with poly-D-lysine and vitronectin. Before recording, the MEA chips were moved to the MEA2100 system (MultiChannel Systems) equipped with temperature control and allowed to equilibrate for 10 min before recording. The data were acquired using Multi Channel Experimenter or Multiwell Screen (MultiChannel Systems) at a sampling rate of 20 kHz for 2 min at 37 °C. Data were filtered using Butterworth band pass filter with 200 Hz cutoff frequency and threshold of 5 x SD were set to minimize both false-positive and missed detection. The representative electrodes were selected for analysis of mean spike frequency and percentage of spikes in the burst.

### Transcriptome analysis

DAVID Functional Annotation Bioinformatics Microarray Analysis can be accessed at https://david.ncifcrf.gov. The raw transcriptomic data of D0-D28 significant DEGs (*p* < 0.05) included 2242 upregulated and 1438 downregulated terms, available in the supplemental material. Each list of *Homo sapiens* genes was independently analyzed by DAVID, to generate an analysis of associated gene ontology (GO) terms.

OPaver (Li, unpublished), is a web-based tool to integrate multiple types (e.g. transcriptomics, proteomics and metabolomics) and time series of data to KEGG biochemical pathways maps. This software analysis tool was developed at Los Alamos National Laboratory. In this case, OPaver was utilized to map significantly differentially expressed genes (*p* < 0.05) identified in the DEseq2 analysis performed by PiRet. Differential expression calculation from DEseq2 in Log2 fold change and associated genes (provided in the supplementary material) were used as input for the OPaver software.

## Supplementary Information

**Additional file 1. **List of differentially expressed genes (DEG) at each stage of MN development that were found to be significantly (*p* < 0.001) up or downregulated compared to the transcriptome profile of iPSC (D0). Additionally, DEG between two consecutive cell types are included.

**Additional file 2. **Snapshot of the Ontology Pathway at the Glutamatergic synapse showing regulatory genes in cAMP and Ca^2+^ signaling pathways with DGE during the transition from iPSC to NSC, MNP (D13 vs D07), early MNs (D18 vs D13) and mature MNs (D28 vs D13). From left to right the color-coded legend shows DGE (*p* < 0.001) upon transition from iPSC to MNs. OPaver software was used to link the DGE profiles to the metabolic pathways.

## Data Availability

The datasets generated for this study can be found in the NCBI SRA database under accession numbers SRR11994167- SRR11994181, NBCI BioProject PRJNA638768, [https://www.ncbi.nlm.nih.gov/bioproject/PRJNA638768] and NCBI BioSamples SAMN15207814-SAMN15207828.

## Abbreviations

ACh: Acetylcholine
ALS: Amyotrophic lateral sclerosis
AP: Action potentials
BDNF: Brain-derived neurotrophic factor
βIII-Tub: Class III β-tubulin
BMP: Bone morphogenic protein
cAMP: cyclic adenosine monophosphate
Cavs: Voltage-dependent calcium-channels
ChAT: Choline acetyltransferase
CHIR: CHIR99021
CNTF: Ciliary neurotrophic factor
CpdE: Compound E
D0: Day 0
D7: Day 7
D13: Day 13
D18: Day 18
D28: Day 28
D31: Day 31
DAVID: Database for annotation, visualization and integrated discovery
DEGs: Differentially expressed genes
eMNs: Early motor neurons
GO: Gene ontology
GPCR: G protein-coupled receptors
HVA: High-voltage activated
IP3: Inositol trisphosphate
iPSC: Induced pluripotent stem cells
IGF-1: Insulin-like growth factor 1
Isl1: Islet-1
LVA: Low-voltage activated
LANL: Los Alamos national laboratory
mAChRs: Muscarinic acetylcholine receptors
MAP 2: Microtubule-associated protein 2
MEA: Multi-electrode array
MN: Motor neurons
MNPs: Motor neuron progenitors
nAChR: Nicotinic acetylcholine receptor
NMJs: Neuromuscular junctions
NSCs: Neural stem cells
OPaver: Ontology pathway analysis software
PCA: Principal component analysis
PLC: Phospholipase C
Pur: Purmorphamine
RA: All-trans retinoic acid
RNA-Seq: RNA-sequencing
SB: SB431542
Shh: Sonic hedgehog.
TGFβ: Tumor growth factor-β
