# Supplementary figures and images for: Global transcriptome profile of the developmental principles of in vitro iPSC-to-motor neuron differentiation
Source: BMC Mol Cell Biol. 2021 Feb 18;22:13. doi: 10.1186/s12860-021-00343-z (PMC7893891; doi:10.1186/s12860-021-00343-z)

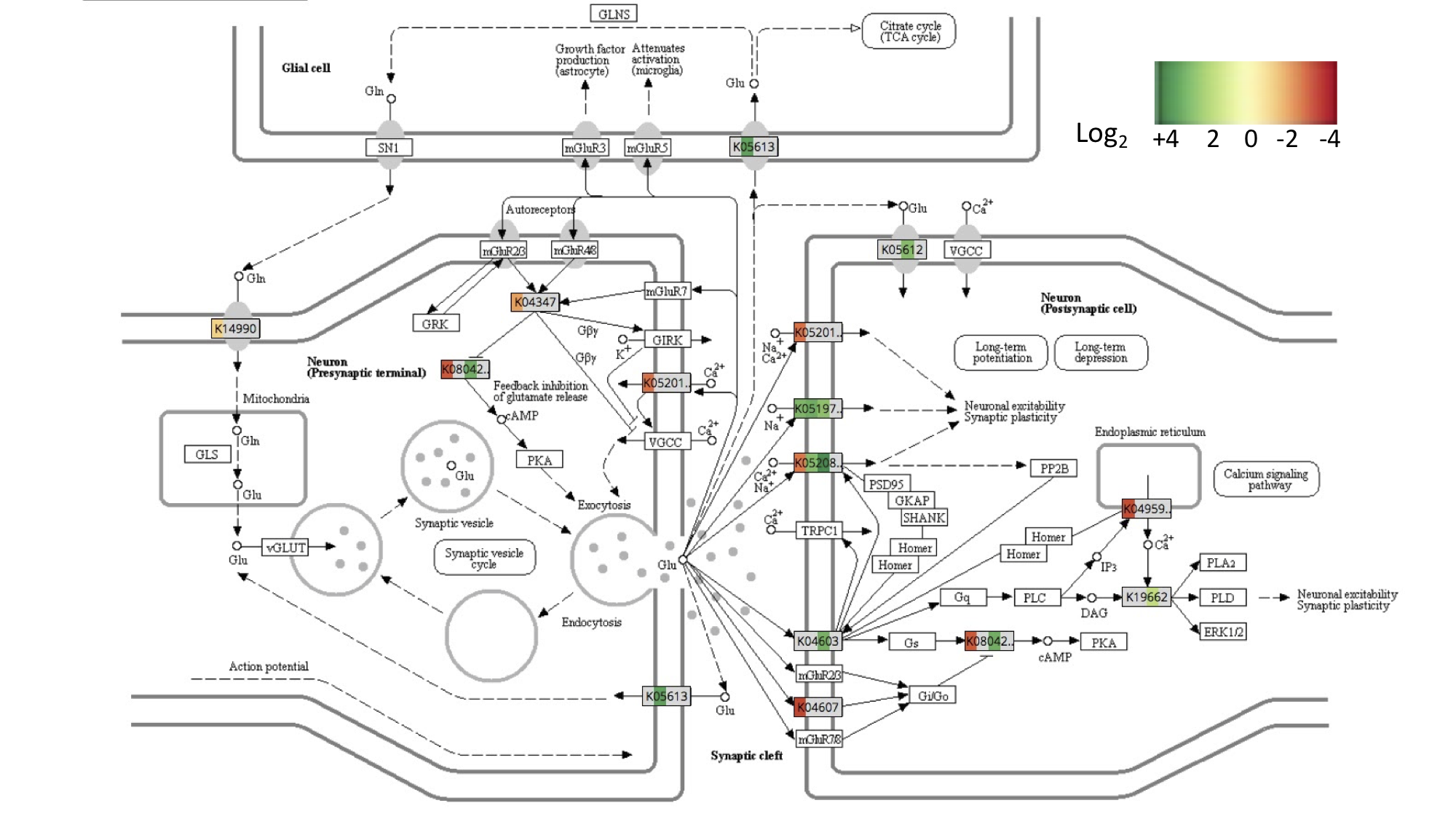

Supplement: Supplementary file 2 — Additional file 2. Snapshot of the Ontology Pathway at the Glutamatergic synapse showing regulatory genes in cAMP and Ca2+ signaling pathways with DGE during the transition from iPSC to NSC, MNP (D13 vs D07), early MNs (D18 vs D13) and mature MNs (D28 vs D13). From left to right the color-coded legend shows DGE (p < 0.001) upon transition from iPSC to MNs. OPaver software was used to link the DGE profiles to the metabolic pathways. [file 12860_2021_343_MOESM2_ESM.tiff]
